# Negative Mood States Correlate with Laterobasal Amygdala in Collegiate Football Players

**DOI:** 10.1155/2018/8142631

**Published:** 2018-02-08

**Authors:** Han Byul Cho, Charles Elliott Bueler, Jennifer DiMuzio, Charlie Hicks-Little, Erin McGlade, In Kyoon Lyoo, Deborah Yurgelun-Todd

**Affiliations:** ^1^The Brain Institute, University of Utah, 383 Colorow Drive, Salt Lake City, UT 84108, USA; ^2^Diagnostic Neuroimaging Lab, University of Utah, 501 Chipeta Way, Salt Lake City, UT 84108, USA; ^3^Department of Psychiatry, School of Medicine, 501 Chipeta Way, Salt Lake City, UT 84108, USA; ^4^Department of Physical Therapy and Athletic Training, University of Utah, 520 Wakara Way, Salt Lake City, UT 84108, USA; ^5^Rocky Mountain MIRECC, Department of Veterans Affairs, 500 Foothill Drive, Salt Lake City, UT 84148, USA; ^6^Ewha Brain Institute and Department of Brain and Cognitive Sciences, Ewha Womans University, 150 Bugahyeon-ro, Seodaemun-gu, Seoul 03759, Republic of Korea; ^7^Graduate School of Pharmaceutical Sciences, Ewha Womans University, 52 Ewhayeodae-gil, Seodaemun-gu, Seoul 03760, Republic of Korea

## Abstract

A number of studies have suggested that sports-related concussion (SRC) may place individuals at increased risk for depression and negative outcomes including suicide. However, the mechanisms underlying a potential relationship between brain integrity and mood remain unclear. The current study is aimed at examining the association between amygdala shape, mood state, and postconcussion symptoms in collegiate football players. Thirty members of 1 football team completed the Profile of Mood States (POMS), the postconcussion symptom scale (PCSS), and an MRI protocol during preseason camp. T1-weighted images were acquired and three-dimensional amygdala and probabilistic maps were created for shape analysis. Correlation analyses between POMS and PCSS and the relationship between POMS and amygdala shape were completed. In the amygdala, the left laterobasal subregion showed a positive relationship with the POMS total score and subscales scores. No significant relationship between PCSS and amygdala shape was found. Significant positive correlations were found between POMS subscales and PCSS. These results indicate that amygdala structure may be more closely associated with negative mood states than postconcussion symptoms. These findings suggest that premorbid individual differences in effect may provide critical insight into the relationship between negative mood and outcomes in collegiate football players with SRC.

## 1. Introduction

Concussion is a type of mild traumatic brain injury (mTBI) that can cause headaches, fatigue, sadness, and nausea (http://www.ncaa.org/health-and-safety/concussion-guidelines). Repeated concussions have been considered a risk factor for Alzheimer's disease and chronic traumatic encephalopathy (CTE) [1, 2]. According to Langlois and colleagues, about 1.6 to 3.8 million concussions occur annually in sports and recreational activities [3]. However, there are also many benefits of sports involvement such as improved physical development, instilling the value of a healthy lifestyle, the promotion of psychological well-being, improved self-esteem, the development of personal responsibility, and improved academic performance [4]. Moreover, the prospect of scholarships incentivizes many athletes to play sports at the collegiate level (http://www.collegescholarships.org/athletic.htm; http://www.ncaa.org/student-athletes/value-college-sports). Opportunities to play sports in college are limited, which can create a very competitive environment. In particular, college football is a contact sport that involves intense competition, both externally against other teams and internally against fellow teammates. Thus, exposure to concussion and head impacts occurs during football practice as well as during the game.

Previous studies with athletes have reported the effects of sports-related concussion on structural brain changes, mood states, and neuropsychological function. A study on professional fighters found that repeated head trauma was associated with lower brain volumes and processing speed [5]. Other investigations have reported a relationship between sports-related concussion and negative mood, depression, and anxiety symptoms [6–9]. However, the mechanism for these mood and cognitive changes and the relationships between brain structural traits, mood states, and postconcussion symptoms in sports-related concussion has not yet been identified.

In order to better understand the relationship between mood states and postconcussion symptoms in sports-related concussion, particular attention should focus on the amygdala and its subregions in individuals at risk for sports-related concussion.

The amygdala has been shown to be functionally related to stress response [10], emotional behavior, and psychiatric conditions [11]. This brain region is composed of multiple subregions that can be divided into three groups including superficial, centromedial, and laterobasal subregions [12, 13]. The subregional groups refer to the different anatomical and functional connectivity between other subcortical brain regions and the cortex [12, 13]. Evidence from previous neuroimaging studies has suggested that differences in amygdala structure are related to concussions or mTBI [14, 15]. Using diffusion tensor imaging, McAllister and colleagues examined the association between exposure to repetitive concussive head impacts in collegiate sports players and the amygdala [14]. Depue and colleagues found differences in amygdala volume and amygdala shape in veterans with PTSD/mTBI group [15]. Despite previous research studies using MRI to assess the impact of concussive and subconcussive episodes on the brain, none have yet explained the relationship between mood states, postconcussion symptoms, and amygdala structure at the detailed level of amygdala subregions. Furthermore, few studies have examined behavioral changes in light of amygdala subregions as measured by shape analysis.

Within the amygdala, there are three subregions. The superficial subregion is known to be related to the olfactory system, emotional processing, and selective social stimuli processing [16–19]. The centromedial subregion is associated with an endocrine response to fearful stimuli and reproductive behavior [16, 19]. The laterobasal amygdala subregion is associated with emotional memory process, regulation of anxiety stimuli, and stress response [16, 17, 19, 20]. These functional differences in subregions highlight the importance of examining shape related changes in amygdala morphometry. The purpose of our study is to describe the association between the structure of the amygdala subregions, mood state, and postconcussion symptoms in collegiate football players.

## 2. Materials and Methods

### 2.1. Subjects

Thirty male subjects between the ages of 18 and 25 years participated in the study. All subjects were student athletes at the University of Utah who participated in the fall football season. They were recruited following the completion of spring football activities and prior to the initiation of fall football camp. All of subjects had had no direct concussion for at least 3 months before they participated in the study. Demographic data is presented in Table 1. Exclusion criteria included any contraindication to MRI, including braces, ferromagnetic metal implants, and unapproved surgical devices. No one was excluded from the study based on these exclusion criteria. All subjects provided written informed consent approved by the Institutional Review Board (IRB) of the University of Utah.

### 2.2. Assessments

The subjects' mood states were measured using the Profile of Mood States (POMS) [21]. The POMS consists of 65 items rated on a five-point Likert-like scale (0, not at all; 1, a little; 2, moderately; 3, quite a bit; and 4, extremely frequently) to yield six subscale scores: tension, depression, anger, fatigue, confusion, vigor, and a total mood disturbance (TMD). High scores for tension, depression, anger, fatigue, confusion, and TMD reflect a negative mood state, and high scores of vigor reflect a positive mood state. All 30 subjects completed the POMS (at baseline). Fourteen of the 30 subjects were available one year later to participate in the follow-up visit (mean follow-up period days were 364.93 ± 4.71). This second data time point was collected to estimate the stability of the POMS score.

The subjects' postconcussion symptom scores were measured using the Immediate Postconcussion Assessment Testing (ImPACT) battery [22]. The postconcussion symptom scale (PCSS) includes 22 items on a six-point Likert-like scale to describe the severity of postconcussion symptoms (0, not experiencing the symptom; 1-2, minor; 3-4, moderate; 5-6, severe). The ImPACT also yields a total symptom composite score of PCSS, which is the sum of the 22 response scores for the 22 items. A total of 29 out of 30 subjects completed the ImPACT PCSS at baseline. It is of note that all individuals who completed the PCSS were concussion-free for at least 3 months before spring football activities; nevertheless some football players reported symptoms. The follow-up PCSS measure was not completed because of participant availability.

### 2.3. Magnetic Resonance Image Acquisition

Brain MRI data were obtained using a 3.0 Tesla Siemens Magnetom Verio scanner. A standard 12-channel head coil was employed for data acquisition. Using a T1-weighted 3D MPRAGE GRAPPA sequence, the axial plane T1-weighted images were acquired with the following parameters: Echo Time (TE) = 3.42 ms, Repetition Time (TR) = 2000 ms, Inversion Time (TI) = 1100 ms, Flip Angle = 8°, 256 × 256 acquisition matrix, 160 slices, and 1.0 mm slice thickness. The image data were transferred from the scanner in DICOM format and coded. A total of 30 subjects completed the MRI scan. The MRI data of each participant were securely examined by a board-certified neuroradiologist to screen gross pathology; no one was excluded based on these evaluations.

### 2.4. Amygdala Segmentation

All segmentations of amygdala structure from all 30 subjects' T1-weighted images were performed using FreeSurfer 5.3.0 [23] (http://surfer.nmr.mgh.harvard.edu/). FreeSurfer pipeline was used to perform motion correction, intensity normalization, skull stripping, Talairach transformation, and gray and white matter segmentation on the T1-weighted images [23, 24]. Subcortical structures, including the amygdala, were automatically labeled and segmented using a probabilistic algorithm [23, 25]. The accuracy of this procedure was verified through comparison with manual trace labeling [23]. The extracted amygdala images were visually inspected by an expert. After extracting the amygdala structural images, they were converted to binary images for shape analysis. Estimated total intracranial volume (eTIV) and amygdala volume also were measured by FreeSurfer processing [23, 26].

### 2.5. Amygdala Shape Analysis

The 2D slices of the binary amygdala images were treated to the 3D amygdala surface by a marching cube algorithm [19, 27, 28]. For the smoothing of the 3D amygdala surface, a 3D Laplacian smoothing algorithm was employed [19, 28]. Using the spherical coordinate points calculated from a principal component analysis (PCA), 30 subjects' amygdalae were aligned and one thousand points were distributed equally on the aligned amygdala surface [28]. Each process was visually inspected and manually corrected to ensure that nothing had been misaligned. Of 60 left and right amygdala images, 10 images processed manual PCA alignment corrections [28]. The radii of the amygdala calculated the distance from the center of inertia (COI) to each surface point. Then the left and right amygdala templates were produced by the averaged radii of each point [19, 28]. Individual amygdala images were registered to the amygdala template to customize this research and improve accuracy. An iterative closest point algorithm [29] was employed for this registration [19, 28].

### 2.6. Amygdala Probabilistic Map

The process to generate probabilistic maps for each amygdala subregion structure reference was consistent with previously reported methodology [19, 28]. We referred to Amunts and colleagues' work on the stereotaxic probabilistic maps of left and right amygdala subregions, which were based on cytoarchitectonic characteristics [13]. The calculated T1-weighted images voxel coordinates matched to 1,000 points on the amygdala surface using PCA and an ICP algorithm. Matrices were calculated by registering T1-weighted images to MNI152 template space using FMRIB's Linear Image Registration Tool (FLIRT) (https://fsl.fmrib.ox.ac.uk/fsl/fslwiki/FLIRT). The matrices were reversed and changed to inverse matrices. The inverse matrices were used to transform Amunts' amygdala subregion probabilistic maps to T1-weighted images native space [19, 28]. We then calculated the probabilities of each amygdala's surface points from each subregion using the transformed Amunts' amygdala subregion probabilistic maps [19, 28]. The probability that the surface points of the amygdala template belong to each subregion was calculated by averaging the probability of each subject's amygdala surface point [19, 28].

**Figure 1:**
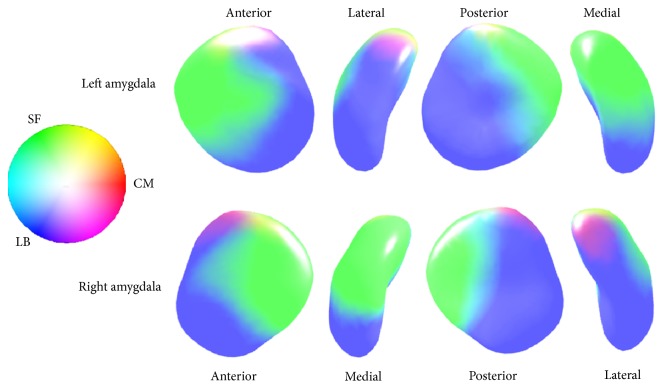
The amygdala probabilistic maps show the laterobasal, superficial, and centromedial subregion on the amygdala template (blue-tone color: laterobasal subregion, LB; green-tone color: superficial subregion, SF; red-tone color: centromedial subregion, CM).

The left amygdala was comprised of 50% laterobasal subregion, 38% superficial subregion, and 12% centromedial subregion. The right amygdala was comprised of 52% laterobasal subregion, 39% superficial subregion, and 9% centromedial subregion in our amygdala templates. Surface probabilistic maps of our amygdala template were visualized as Figure 1.

### 2.7. Statistical Analysis

Linear regression was used to analyze the relationship between the POMS subscales and amygdala volume and the relationship between POMS subscales and adjusted mean radii for significant cluster. Generalized linear models were used to analyze the relationship between the POMS subscales and radii from the COI and the ImPACT PCSS and radii from COI. Pearson correlation was used to analyze the relationship between the POMS subscales and ImPACT PCSS score. Mann–Whitney *U* test was used to compare ImPACT PCSS score and amygdala volume between the concussion ≥ 1 subjects and nonconcussed subjects. Linear mixed-effects model was used to compare POMS scores of baseline and follow-up. The period between baseline and follow-up was included as fixed effects, and subjects were included as random effects. Age and eTIV were included as covariates for linear regression analysis and the generalized linear models analysis and used to adjust the mean radii for amygdala shape analysis' significant cluster. Statistical significance was defined at alpha < 0.05. For the amygdala shape analysis, the false discovery rate (FDR) method was employed to correct for multiple comparisons [30]. As an additional step to reduce the possibility of false-positive findings, clusters with cluster sizes smaller than 15 surface points were eliminated [31]. In addition, we used a linear mixed-effects model to compare POMS scores at baseline and follow-up. The period between baseline and follow-up was the fixed effect, and subjects were the random effect. All statistical analyses were performed with STATA version 12.1 (StataCorp, College Station, Texas, USA).

## 3. Results

### 3.1. Demographic, Clinical Information and Assessments

Subjects' demographic and clinical information is summarized in Table 1. Subjects included 30 male football players ranging from 18 to 25 years of age (mean age = 21.20 ± 1.58). Very few subjects reported having sustained concussions during the prior football seasons. Specifically, 19 participants reported having sustained 0 concussions, 6 subjects reported 1 concussion, and 5 subjects reported 2 concussions. Interestingly, subconcussive symptoms measured by the ImPACT were more frequently reported.

Correlations between the POMS subscale scores and ImPACT PCSS score are summarized in Table 2. There were significant positive correlations between ImPACT PCSS score and POMS depression (*r* = 0.39, *p* = 0.037), POMS anger (*r* = 0.37, *p* = 0.043), POMS fatigue (*r* = 0.48, *p* = 0.008), POMS confusion (*r* = 0.50, *p* = 0.006), and POMS TMD (*r* = 0.43, *p* = 0.021). There were no significant differences between POMS scores at baseline and 1-year follow-up (Table 3). There was no significant difference in ImPACT PCSS between players with a history of concussion (concussion ≥ 1) and those who reported no concussion.

### 3.2. Amygdala Shape Analysis

**Figure 2:**
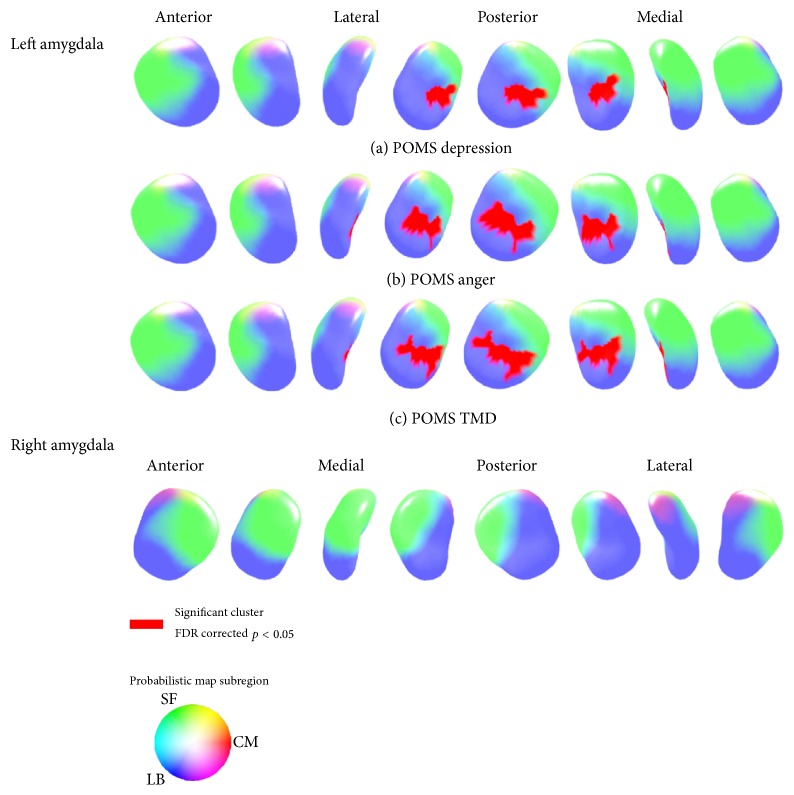
The amygdala area shows the positive relationships between POMS subscales and amygdalar radii. Significant positive associations were found between the amygdala radii and POMS subscales, including (a) POMS depression, (b) POMS anger, and (c) POMS total mood disturbance (TMD). There was no area of significant negative association (false discovery rate [FDR] corrected *p* < 0.05, cluster surface points > 15). (POMS, Profile of Mood States; TMD, total mood disturbance; LB, laterobasal subregion; SF, superficial subregion; CM, centromedial subregion).

**Figure 3:**
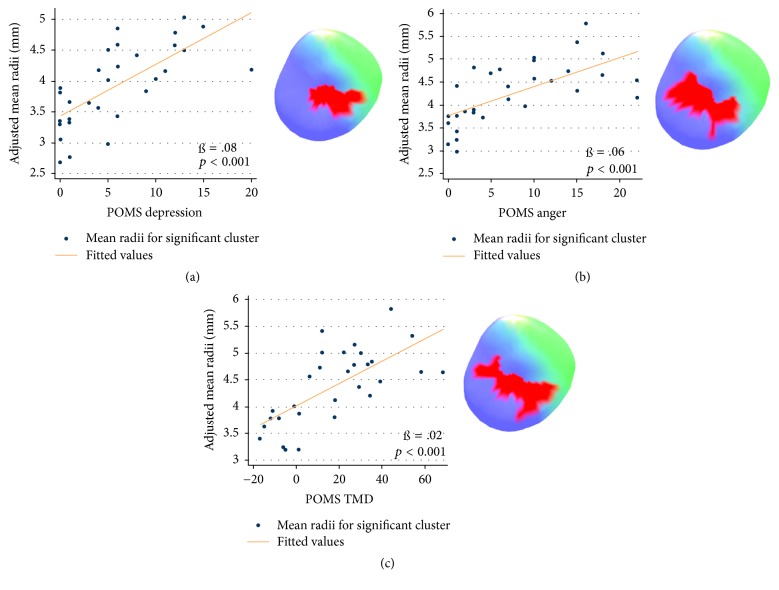
The graphs with scatter plots explain the relationship between POMS subscales and adjusted mean radii for significant cluster. The mean radii were adjusted for age and estimated total intracranial volume (eTIV). The relationship between POMS depression (a), POMS anger (b), POMS total mood disturbance (TMD) (c), and the adjusted mean radii as shown.

The radii of the left laterobasal amygdala subregion had a positive relationship with the POMS depression subscale (cluster surface points 101), POMS anger subscale (cluster surface points 125), and POMS TMD score (cluster surface points 118) after including age and eTIV as covariates. As can be seen in the amygdala probabilistic maps, the significant clusters were in left laterobasal amygdala subregion (Figure 2). Specifically, the cluster's mean radii showed a positive relationship with the POMS depression (ß = 0.08; *t* = 5.11; *p* < 0.001), anger (ß = 0.06; *t* = 4.54; *p* < 0.001), and TMD scores (ß = 0.02; *t* = 4.94; *p* < 0.001) (Figure 3). There were no significant relationships evident between the amygdalar radii and the POMS tension, POMS fatigue, POMS confusion, and POMS vigor subscales.

There was no significant relationship between the amygdalar radii and the score on the ImPACT PCSS when the entire group of athletes was examined.

**Figure 4:**
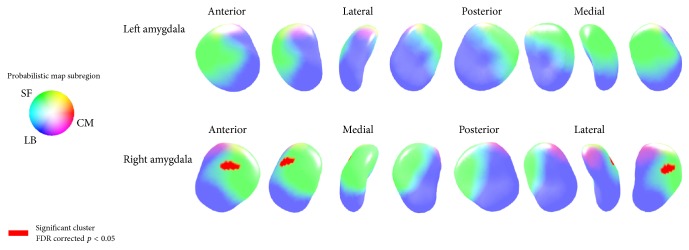
The amygdala area shows the positive relationships between PCSS and amygdalar radii in concussion ≥ 1 subjects. The significant cluster of right amygdala association between amygdala radii and PCSS. There was no area of significant negative association (false discovery rate [FDR] corrected *p* < 0.05, cluster surface points > 15). (PCSS, postconcussion symptom scale; TMD, total mood disturbance; LB, laterobasal subregion; SF, superficial subregion; CM, centromedial subregion).

**Figure 5:**
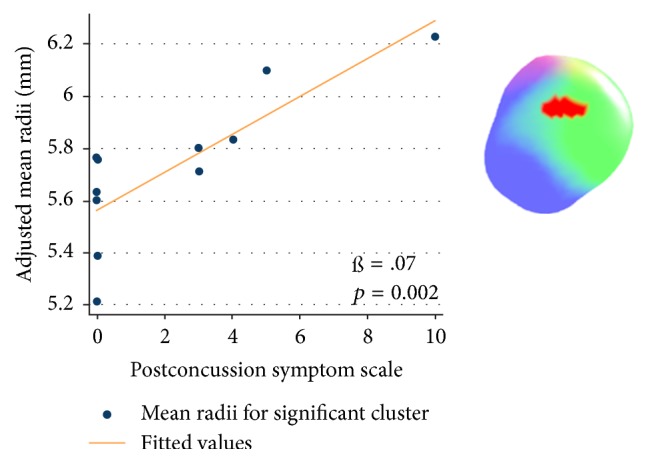
The graphs with scatter plots explain the relationship between PCSS and adjusted mean radii for significant cluster in concussion ≥ 1 subjects. The mean radii were adjusted for age and estimated total intracranial volume (eTIV).

However, the radii of the right superficial subregion showed a positive relationship with the ImPACT PCSS in players with a history of concussion (cluster surface points 26) (Figure 4). The significant cluster's mean radii showed positive relationship with the ImPACT PCSS in players with a history of concussion (ß = 0.07; *t* = 4.20; *p* = 0.002) (Figure 5).

### 3.3. Amygdala Volume Analysis

The linear regression analysis found no significant relationship between POMS subscales and amygdala volume or ImPACT PCSS score and amygdala volume after including age and eTIV as covariates. There was no significant difference in amygdala volume after including age and eTIV as covariates between players with a history of concussion and those who reported no concussion.

## 4. Discussion

The current study found a significant relationship between the left laterobasal amygdala subregion and negative mood states in collegiate football players. To the best of our knowledge, this is the first study to examine the relationship between amygdala shape and negative mood states in collegiate sports players. The laterobasal amygdala subregion includes the lateral nucleus and the basolateral, basomedial, and paralaminar nuclei of the amygdala [12, 13, 16]. This amygdala subregion is known to be associated with emotional memory, motor response to fear stimuli, anxiety [16, 17], and stress response [10]. Similar to our findings, prior research has shown a strong relationship between the amygdala and emotion, stress, depressive symptoms, and anxiety. Specifically, previous studies have reported structural amygdala abnormalities in patients with clinical mood disorders [32–34]. One recent study by Rubinow reported an increased number of neurovascular cells in the basolateral amygdala in individuals with major depressive disorder based on a postmortem tissue analysis [35]. Additionally, Joshi and colleagues reported that the basolateral nucleus of the amygdala expanded in response to clinical therapy in major depression [36]. In recent animal studies, the basolateral amygdala nuclei were correlated with stress and stress-related neurotransmission [10] and enhanced fear learning under the stressful postbrain injuries [37].

While some previous findings are not consistent with the current results [34, 36], it is important to consider that those studies focused on clinical subjects who have diagnosed mood disorders according to the DSM-IV or DSM-IV TR, while the current study focused on nonclinical college athletes as exclusion criteria for all subjects include any DSM-IV mental disorders. Additionally, the POMS scale used in our study is a more robust measure of transient, fluctuating feelings, and enduring mood states rather than direct clinical mood disorder symptoms [21]. Therefore, the current finding of a positive relationship between the laterobasal amygdala subregion and negative mood state scale suggests an association between this limbic region and negative mood [38]. Furthermore, studies relying on amygdala volume may differ in their results when compared with studies using an amygdala shape method [19, 28]. It should be noted that the current findings do not address whether amygdala shape is impacted by specific competitive conditions or the stress that football players may have experienced.

The study results showed a positive correlation between the left laterobasal amygdala subregion and POMS depression, anger, and TMD; however, only the left laterobasal amygdalar radii were associated with negative mood states. These findings might be caused by the lateralization of amygdala function. Although there has been little research conducted on the structural lateralization of amygdala subregions, previous functional MRI studies have reported lateralized amygdala activation to a variety of stimuli [39–47]. Reports suggest that the left amygdala is involved in the enhancement of stimuli awareness [39], cognitive representation of fear arousal [40], and activation of negative emotion processing [45, 47]. Compared to the right amygdala, the left amygdala is more involved in processing specific, sustained, and detailed emotional stimuli [41, 43]. The right amygdala is involved in global emotional processing [44], faster habituation response [41, 42], and automatic response for emotional stimuli [43]. The current study extends prior research on the lateralization of the amygdala by reporting the relationship between the specific negative mood states in collegiate football players (i.e., competitive situation). The correlation between the left amygdala laterobasal subregion and negative mood states could suggest that negative mood states in collegiate football players are related more to detailed emotional processing than to automatic/habituated emotional response.

There were no significant correlations between amygdala shape or amygdala volume and PCSS. We observed a positive relationship with negative mood states for the entire group of participants, whereas we observed a positive relationship between right amygdala superficial subregion and PCSS in the athletes who had a history of concussion. A previous study with comorbid PTSD/mTBI veterans demonstrated a reduction of anterior amygdala volume and shape compared to veterans with no history of mTBI, such that greater volumetric reductions in the amygdala relate to cognitive deficits and greater PTSD symptoms [15]. Additionally, a previous study using diffusion tensor imaging to study a group of nonconcussed collegiate sports players found an association between exposure to repetitive head impacts and changes in DTI measures in the amygdala [14]. However, these previous studies lacked direct evidence that postconcussion effects related to the structure of the amygdala because the study design did not focus directly on postconcussion symptoms or sports-related concussion. Neither the current study nor previous studies can fully explain the relationship between amygdala structure and postconcussion symptoms, since it is possible that the observed structural change in the laterobasal amygdala subregion may be influenced more by negative mood states than by postconcussion symptoms. Additionally, the finding of a positive relationship between right amygdala superficial subregion and PCSS in the concussed subjects may imply that the superficial amygdala subregion may relate more to the control, sensing, or emotional processing associated concussion rather than to structural damage.

There was a significant positive correlation between PCSS and negative mood states in collegiate football players. This is particularly interesting considering that when the PCSS were measured, subjects had been concussion-free for at least 3 months before spring football activities. Moreover, current subjects had lower mean PCSS (2.41 ± 4.97) than the cutoff value of 7 for diagnosis of concussion [48]. Thus, the abovementioned correlation may be seen as a reflection of the impact of contact sports per se on collegiate players' well-being, not the direct impact of concussion. Nevertheless, these results are similar to those of previous studies that reported a relationship between postconcussion symptoms and negative mood states or depression symptoms [6–8, 49]. It is also possible that the negative mood states and postconcussion symptoms in collegiate football players might be related to the characteristics of competitive collegiate football [7, 50]. The subjects of the current study were members of the University of Utah football team, which is in the Pac-12 (http://pac-12.com/). Therefore, subjects had exposure to high levels of competition and potentially stressful situations during the football season.

The incidence of concussion in collegiate football players has been higher than that of high school and youth football players [50]. It has also been reported that collegiate athletes have more emotional and sleep symptoms as a result of their concussions than high school athletes [51]. These symptoms may reflect the competitive situation that collegiate athletes experience [50]. The current results and those of previous studies may suggest additional indirect effects of competition collegiate football games and concussion exposure. On the other hand, the lack of specific postconcussion symptoms in concussed individuals and/or mTBI groups has been a point of discussion [52, 53]. Iverson reported that chronic traumatic encephalopathy in athletes could result from a comorbid condition and not represent a major risk factor for depression and/or suicidality [54]. Results from previous studies as well as our own cannot address the causative relationship between sports-related concussion symptoms and negative mood symptoms in collegiate athletes conclusively. Findings from the current study are consistent with the perspective that the competitive environment of collegiate football players could lead to increased concussion exposure, postconcussion symptoms, and negative mood concurrently.

We also found that the collegiate football players' negative mood states were stable 1 year following their initial preseason assessment, although a subsample completed follow-up ratings. Previous studies reported that the sports players' negative mood states have been higher in the precompetitive game situation compared to the postcompetition game or postmatch rest period [55, 56]. Our subjects' POMS were measured twice with a 1-year interval, in consecutive preseason period. Taking that into account, it seems that the collegiate football players' negative mood states may have been affected by preparation for a competitive season rather than a consequence of a latent accumulation of concussion exposure.

The current study demonstrates an association between amygdala shape and negative mood states; however, amygdalar shape changes were not associated with postconcussion symptoms. Findings also show a positive relationship between negative mood states and postconcussion symptoms in collegiate football players. These results suggest that structural change in the amygdala subregion is more closely related to negative mood than postconcussion symptoms in collegiate athletes. These results provide insight into potential mechanisms for the relationship between postconcussion symptoms, negative mood, and the amygdala in individuals with sports-related concussion.

It should be noted that approximately 1/3 of football players included in this investigation reported a history of concussion in the prior season, suggesting that study findings may be related to factors other than concussive injury. Thus some of the findings may not generalize to groups that are more homogeneous with regard to concussion history. Furthermore, only football players were included in this study so it is not possible to conclude that the observed findings are related specifically to football players as opposed to other collegiate students.

## 5. Limitations and Future Directions

The current study was cross-sectional, making it difficult to establish causal relationships between amygdala shape, PCSS, and mood symptoms. This highlights the need for future studies including longitudinal data to elucidate the mechanisms by which the structure of the amygdala, mood, and concussive experiences interact. Future research will benefit from studies including control groups to examine amygdala, mood, and PCSS differences between groups in addition to interactive differences between these factors. Finally, the current study included only males. While this is an advantage in controlling for sex effects, generalizability across sexes is also limited.

## 6. Conclusions

The current study found a significant positive relationship between the laterobasal amygdala and negative mood states in college football players using a shape analysis technique. An association was also seen between postconcussion symptoms and negative mood states although concussion symptoms were not correlated with amygdalar shape. These results suggest that amygdala structure may be more closely associated with negative mood states than postconcussion symptoms and imply that premorbid individual differences in effect may provide critical insight into the relationship between negative mood and outcome in collegiate players with sports-related concussion.

## Figures and Tables

**Table 1:** Demographic information, clinical characteristics, and volume measurements of study subjects.

	Football players *N* = 30
*Demographic information*	
Age (year), mean (SD)	21.69 (1.58)
Gender (male/female)	30/0

*Clinical characteristics*	
*POMS*, mean (SD)	
Tension	8.70 (4.84)
Depression	5.90 (5.37)
Anger	7.87 (6.94)
Fatigue	6.67 (4.79)
Confusion	6.10 (4.39)
Vigor	17.63 (5.57)
TMD	17.60 (22.59)

*ImPACT PCSS*, mean (SD)^¶^	2.41 (4.97)

*Brain volume (mm3)*, mean (SD)	
Left amygdala	1614.57 (197.31)
Right amygdala	1667.62 (184.15)
eTIV (cm^3^)	1466.89 (200.74)

^¶^A total of 29 out of 30 subjects completed the ImPACT PCSS; SD, standard deviation; POMS, Profile of Mood States; TMD, total mood disturbance; ImPACT, Immediate Postconcussion Assessment Testing; PCSS, postconcussion symptom scale; eTIV, estimated total intracranial volume.

**Table 2:** Correlation coefficients between POMS subscales scores and ImPACT PCSS scores.

	POMS						
	Tension	Depression	Anger	Fatigue	Confusion	Vigor	TMD
ImPACT PCSS^¶^	0.19	0.39^*∗*^	0.38^*∗*^	0.48^*∗∗*^	0.50^*∗∗*^	0.12	0.43^*∗*^

^*∗*^ *p* < 0.05; ^*∗∗*^*p* < 0.01. ^¶^A total of 29 out of 30 subjects completed the ImPACT PCSS; POMS, Profile of Mood States; TMD, total mood disturbance; ImPACT, Immediate Postconcussion Assessment Testing; PCSS, postconcussion symptom scale.

**Table 3:** POMS scores of study subjects that were available for follow-up measurement.

POMS, mean (SD)		Football players *N* = 14	
	Baseline	Follow-up (1 year later)	*p*
Tension	9.57 (5.75)	8.57 (7.37)	0.57
Depression	7.57 (6.44)	10.14 (11.50)	0.42
Anger	9.64 (7.44)	10.57 (9.94)	0.68
Fatigue	7.29 (5.72)	7.36 (5.43)	0.98
Confusion	6.71 (5.43)	6.43 (5.15)	0.85
Vigor	19.00 (5.35)	18.29 (5.68)	0.55
TMD	21.79 (27.68)	24.79 (39.79)	0.78

SD, standard deviation; POMS, Profile of Mood States; TMD, total mood disturbance.
